# Selenium Deficiency Exacerbates Hyperoxia-Induced Lung Injury in Newborn C3H/HeN Mice

**DOI:** 10.3390/antiox13040391

**Published:** 2024-03-25

**Authors:** Lora C. Bailey-Downs, Laura G. Sherlock, Michaela N. Crossley, Aristides Rivera Negron, Paul T. Pierce, Shirley Wang, Hua Zhong, Cynthia Carter, Kathryn Burge, Jeffrey V. Eckert, Lynette K. Rogers, Peter F. Vitiello, Trent E. Tipple

**Affiliations:** 1 University of Oklahoma Health Sciences Center, Oklahoma City, OK 73104, USA; lora-bailey-downs@ouhsc.edu (L.C.B.-D.); xiu-wang@ouhsc.edu (S.W.); hua-zhong@ouhsc.edu (H.Z.); cynthia-carter@ouhsc.edu (C.C.); kathryn-burge@ouhsc.edu (K.B.); lynette-rogers@ouhsc.edu (L.K.R.); peter-vitiello@ouhsc.edu (P.F.V.); 2 University of Colorado Anschutz Medical Campus, Aurora, CO 80045, USA; laura.sherlock@cuanschutz.edu; 3 Oklahoma Children’s Hospital OU Health, Oklahoma City, OK 73104, USA

**Keywords:** selenium, newborn hyperoxia, lung, antioxidants, Wnt/β-catenin, Notch

## Abstract

Extremely preterm infants are often treated with supraphysiological oxygen, which contributes to the development of bronchopulmonary dysplasia (BPD). These same infants exhibit compromised antioxidant capacities due in part to selenium (Se) deficiency. Se is essential for basal and inducible antioxidant responses. The present study utilized a perinatal Se deficiency (SeD) mouse model to identify the combined effects of newborn hyperoxia exposure and SeD on alveolarization and antioxidant responses, including the identification of affected developmental pathways. Se-sufficient (SeS) and SeD C3H/HeN breeding pairs were generated, and pups were exposed to room air or 85% O_2_ from birth to 14 d. Survival, antioxidant protein expression, and RNA seq analyses were performed. Greater than 40% mortality was observed in hyperoxia-exposed SeD pups. Surviving SeD pups had greater lung growth deficits than hyperoxia-exposed SeS pups. Gpx2 and 4 protein and Gpx activity were significantly decreased in SeD pups. Nrf2-regulated proteins, Nqo1 and Gclc were increased in SeD pups exposed to hyperoxia. RNA seq revealed significant decreases in the Wnt/β-catenin and Notch pathways. Se is a biologically relevant modulator of perinatal lung development and antioxidant responses, especially in the context of hyperoxia exposure. The RNA seq analyses suggest pathways essential for normal lung development are dysregulated by Se deficiency.

## 1. Introduction

Prematurity and the consequences of preterm birth remain a significant world-wide health burden. Bronchopulmonary dysplasia (BPD), a chronic lung disease of prematurity, remains the leading cause of respiratory morbidity, mortality, and long-term complications in prematurely born infants, predominantly driven by increases in the survival of extremely low birthweight (ELBW) infants. Preterm neonates are often treated with supraphysiological levels of oxygen (O_2_) to maintain adequate tissue oxygenation; however, hyperoxia and O_2_ toxicity contribute to the development of BPD. These infants are highly susceptible to the effects of O_2_ toxicity due to developmental deficits in endogenous antioxidant defenses [1]. Key antioxidant systems, including the glutathione (GSH) and thioredoxin (Trx) systems, require selenium (Se) for optimal function [2]. Further, GSH system disruption alters lung morphogenesis and hyperoxia responses in neonatal mice [3].

Maternal Se deficiency is associated with adverse pregnancy outcomes including preeclampsia, gestational diabetes, premature delivery, and low birth weight [2,4,5,6,7]. In the fetus, Se is largely trans-placentally acquired during the third trimester. Extremely preterm and extremely low birthweight (<1000 g; ELBW) infants miss third trimester Se accrual and are more profoundly Se deficient [8]. Importantly, ELBW infants were recently shown to be up to 17 times more likely to be Se deficient than term infants [9]. At birth, preterm infants exhibit lower circulating Se levels and lower hepatic Se stores compared to term infants. Standard parenteral and enteral neonatal nutritional protocols lead to insufficient Se supplementation and Se deficiency [4,10]. Thus, perinatal Se deficiency is driven by a lack of Se transfer, is exacerbated by inadequate and/or inconsistent postnatal Se supplementation and is common in ELBW infants [4,11,12,13].

The biological effects of Se are primarily mediated through selenocysteine (Sec), the 21st naturally occurring amino acid. Humans express 25 selenoproteins containing at least one Sec residue. Sec is primarily transported throughout the body via selenoprotein P (Selenop). Selenop contains up to 10 Sec residues and is responsible for ~65% of all circulating Se in humans [14,15]. In catalytic antioxidant selenoproteins, Sec is in the active site and is responsible for oxidation-reduction reactions. Catalytic antioxidant selenoproteins include all isoforms of the glutathione peroxidases (Gpx) and thioredoxin reductases (Txnrd). Optimal Gpx and Txnrd catalytic function are essential for antioxidant responses. Se deficiency has been linked to neurological dysfunction [16] and cancer risks [17,18,19], and high levels of Se is associated with GI morbidities and even death [20]. In settings of limited Se bioavailability, the human body prioritizes the synthesis and expression of specific selenoproteins, a process known as the selenoprotein hierarchy [21]. The selenoprotein hierarchy in the neonatal lung in the setting of altered Se levels and variable oxygen exposure is incompletely characterized.

All Gpx and Txnrd isoforms and the enzyme responsible for de novo GSH synthesis (glutamate-cysteine ligase catalytic unit, Gclc) are transcriptionally regulated by nuclear factor erythroid 2 related factor 2 (Nrf2), the so called “master regulator of antioxidant responses”. Disruptions to GSH-dependent antioxidant responses in the neonatal lung alters lung development and exacerbates hyperoxia-induced lung injury [3]. Thus, the synthesis and activity of Trx and GSH antioxidant systems are modulated by both Se availability and Nrf2 activity [11,22,23]. The studies presented herein were conducted on a C3H/HeN background with a responsive Nrf2 phenotype [24]. We recapitulated the conditions used for our previous studies in C57Bl/6J mice, which utilized a well-established dietary protocol [25]. We established Se-deficient (SeD) male and female breeders and identified the presence of Se deficiency in their newborn pups. We further adapted this model to test the hypothesis that maternal Se deficiency would exacerbate the effects of O_2_ toxicity in association with alterations in pulmonary selenoenzyme expression and function. Further, RNA seq data suggest new pathways affected by the intersection between perinatal Se deficiency and postnatal hyperoxia exposure.

## 2. Materials and Methods

### 2.1. Animal Model

Animal studies were performed at the University of Oklahoma Health Sciences Center using protocols approved by the Institutional Animal Care and Use Committee. C3H/HeN mice were purchased from Harlan (Inotiv, 040, Indianapolis, IN, USA). At 3 weeks of life, male and female mice were randomized to Se-sufficient (SeS) (0.4 ppm sodium selenite from Torula yeast; TD.07326, Inotiv) or Se-deficient (SeD) (<0.01 ppm sodium selenite; TD.92163, Inotiv) diets, and maintained on these diets throughout the experiment. After 3 weeks on respective SeS or SeD diets, mice were bred to create SeS and SeD litters for use in the present studies [25]. Newborn mice were exposed to room air (FiO_2_ 0.21) or hyperoxia (FiO_2_ 0.85), beginning within 12 h of life on postnatal day 0.5 (PND 0.5) for up to 14 d. Dams were rotated between conditions every 24 h to prevent oxygen toxicity. At PN14, pups were euthanized, and the left lung was tied off, removed, snap frozen, and stored at −80 °C for molecular analyses. The right caudal lobe was then inflation-fixed with 10% formalin for morphology. A portion of the animals were euthanized at PN1 or 3 and lungs were perfused with ice-cold PBS, snap frozen in cold TRIzol (15596026, Invitrogen, Waltham, MA, USA), and stored at –80 °C for RNA seq analyses.

### 2.2. Morphometrics

To perform lung morphometry analysis, lungs were gravity inflated under 20 cm fluid pressure with 10% buffered formalin and stored at 4 °C for 24 h. Caudal lobes were paraffin-embedded, cut into 5 µm sections, H&E stained, and brightfield-imaged at 20X (0.33 µm/pixel resolution) using an Olympus slide scanner (VS120-L100-W, Evident Corporation, Shinjuku Monolith, 3-1 Nishi-Shinjuku 2-chome, Shinjuku-ku, Tokyo, Japan). Adobe Photoshop was used to exclude areas of no interest before normalization was performed using the histogram function. ImageJ (v1.54F) and Excel (2024)were used to quantify airspace, alveolar size, mean linear intercept, and radial alveolar count, as adapted from [26,27].

### 2.3. Western Blots

Lung tissues were lysed in 1× RIPA buffer using a BeadBlaster24R (Benchmark Scientific, Sayreville, NJ, USA). Proteins were separated into 4–20% SDS-polyacrylamide gels and semi-dry-transferred to nitrocellulose membranes. Membranes were blocked for 1 h in 5% milk and probed overnight on a 4 °C rocker with the following primary antibodies: anti-Gclc (ab190685, Abcam, Cambridge, UK), anti-Gpx2 (ab137431, 1:2000, Abcam), anti-Gpx4 (sc-166570, 1:1000, Santa Cruz, Santa Cruz, CA, USA), NQO1 (ab34173, 1:2000, Abcam), Sepp1 (PA5-112707, 1:1000, Invitrogen, Waltham, MA, USA) or Txnrd11 (ab124954, 1:1000, Abcam). Membranes were then incubated at room temperature for 1 h in secondary antibody of goat anti-mouse HRP (horseradish peroxidase) (1031-05, 1:2000, Southern Biotech, Birmingham, AL, USA) or goat anti-rabbit HRP (4030-05, 1:2000, Southern Biotech). Membranes were developed using enhanced chemiluminescence (ECL) with ChemiDoc imaging and quantified by densitometry using Image Studio (Li-Cor, v 5.2.1, Lincoln, Nebraska). All quantifications for western blots were normalized to β-actin (8H10D10, 1:2000 Cell Signaling Technology, Danvers, MA, USA).

### 2.4. GPX Activity

Snap-frozen lung tissues (10–15 mg) were homogenized in 300 µL 1× Mammalian Cell Lysis Buffer (ab179835, Abcam) in a BeadBlaster24R (Benchmark) using a linear speed of 4 m/s for two 45 s cycles. Gpx activity (ab219926, Abcam) measurement was based on a series of reactions that ended with a NADP signal that reacted with NADP+ to give a fluorescent signal (420 nm for excitement/480 nm for emission) that was directly proportional to GPx activity.

### 2.5. RNA Sequencing

Mouse lung samples in TRizol (15596026, Invitrogen) were submitted to the Institutional Research Core Facility for processing. RNA was isolated from the tissues using the Direct-Zol RNA miniprep kit and established protocols from Zymo Research (R2050S, Irvine, CA, USA). RNA was checked for quality using Agilent’s 2100 Bioanalyzer (Santa Clara, CA, USA), and concentrations from NanoDrop (ThermoFisher, Waltham, MA, USA) readings were used. Stranded RNA-seq libraries were constructed using a NEBNext poly(A) mRNA isolation kit followed directly by IDT’s XGen Broad Range RNA Library Prep Kit and the established protocols. The library construction was performed using 1 ug of RNA. Each of the 28 libraries was indexed during library construction in order to multiplex for sequencing. Libraries were quantified using Invitrogen’s Qubit 4 fluorometer and checked for size and quality on Agilent’s 2100 Bioanalyzer. Samples were normalized and pooled onto a 150 paired end run on Illumina’s NextSeq 2000 Platform to obtain 50 M reads per sample. Upstream analysis was performed using OSCER HPC (High Performance Computing); this included quality control, read trimming, mapping and read counts and used FastQC v0.12.0, trimmomatic v0.39, HISAT2 v2.2.1 with mouse genome assembly GRCm39 and feature Counts v2.0.6, respectively. DeSeq2 v1.40.2 in R v4.3.1 was used to identify differentially expressed genes with Log2 change > |0.25| and adjusted *p* value < 0.05. Gene set enrichment analysis (GSEA) was conducted with GSEA 4.1.0 against the Hallmark database, and significant pathways were defined as adjusted at *p* < 0.05 and with overlap size > 15.

## 3. Results

### 3.1. Se Deficiency Impact Survival in Hyperoxia

**Figure 1:**
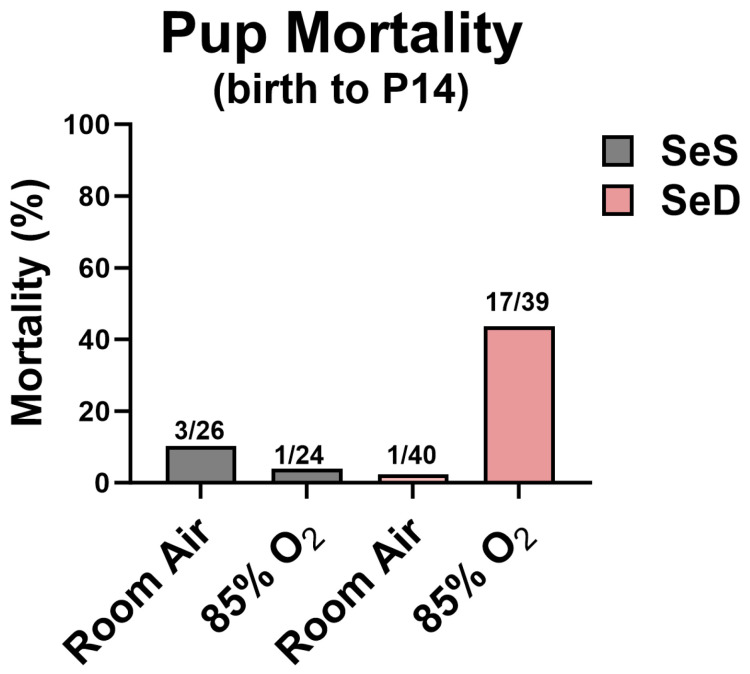
Pup mortality. Pups nursed by dams, fed SeS and SeD diets and exposed to 85% O_2_ for 14 days were observed. Greater than 40% mortality occurred in the SeD, hyperoxia-exposed pups. (Numbers indicate deaths at P14/total live births.).

Pups were reared in either RA or 85% O_2_ from birth to PN14, as described in Methods. The total number of live pups born and the mortality at PN14 are indicated in Figure 1. SeD pups exposed to 85% O_2_ had a higher mortality than SeS pups exposed to hyperoxia or either RA-exposed group (Figure 1). Forty-three percent of pups born to the SeD group and exposed to 85% O_2_ died within the first 14 days of life, predominantly in the second week of hyperoxia exposure.

### 3.2. Morphometric Analysis Reveals Lung-Growth Deficit

**Figure 2:**
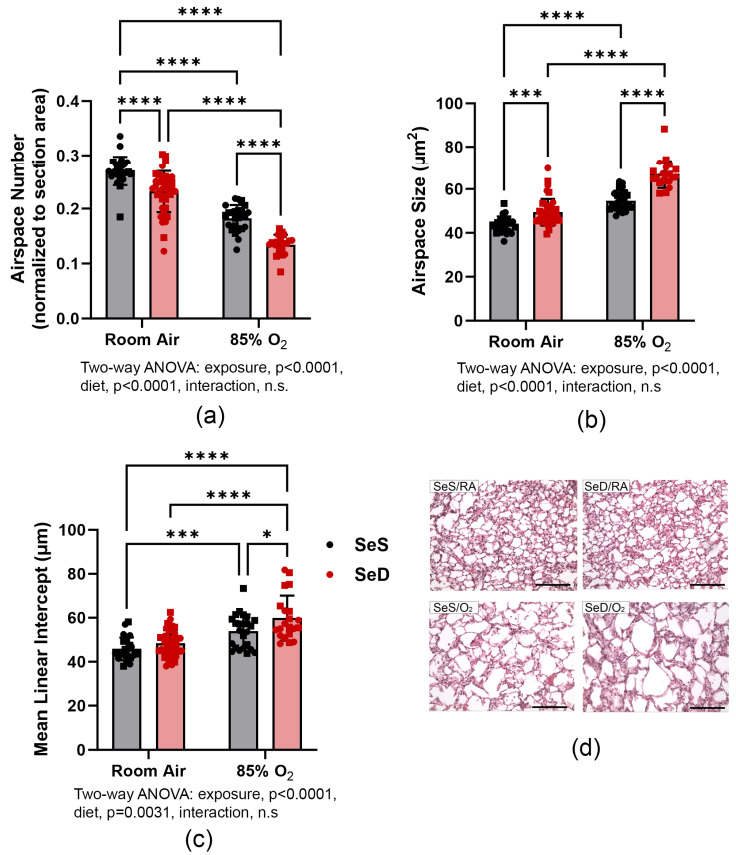
Morphometric analyses. Grey bars with black symbols represent SeS mice while red bars and red symbols represent SeD mice. The alveolar number (**a**) and size (**b**) and mean linear intercept (MLI) (**c**) were measured as described in Section 2. Data were analyzed by two-way ANOVA, with Tukey’s post-hoc analysis. Significance is indicated within the figure: * *p* < 0.05, *** *p* < 0.0005, **** *p* < 0.0001, n.s., non-significant. Se/RA, *n* = 25; SeD/RA, *n* = 40; SeS/O_2_, *n* = 24; SeD, *n* = 22. Photomicrographs of lung tissue sections (**d**) were captured at 200×; bars represent 100 μm. Squares represent males and circles represent females within each treatment group.

Morphometric analyses were performed on H&E-stained lung sections obtained from PN14 pups (Figure 2a–c). Airspace number (a), alveolar size (b) and mean linear intercept (MLI) (c) were determined as described in Methods. An independent effect of hyperoxia on MLI, alveolar size and airspace number was detected. SeD also independently impacted alveolar development, as indicated by alterations in MLI, alveolar size and airspace number (Figure 2). The effects of 85% O_2_ exposure were exacerbated in SeD pups, as evidenced by the greater alveolar size and lower airspace numbers compared to hyperoxia-exposed SeS pups. Photomicrographs of selected histological samples demonstrate the lung-growth deficiencies under SeD and hyperoxia exposure (d).

### 3.3. Gpx Protein Levels and Activity

**Figure 3:**
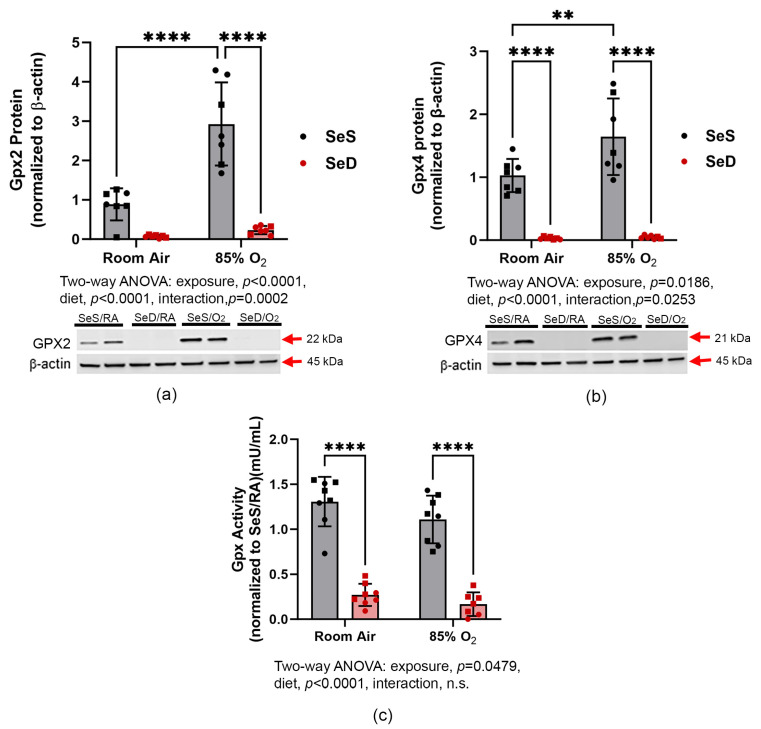
Gpx2 and Gpx4 protein levels and total GPX activity. Gpx2 (**a**) and Gpx4 (**b**) protein levels were measured by western blot analysis, and total Gpx activity (**c**) was measured as indicated in Methods. Grey bars with black symbols represent SeS mice while red bars and red symbols represent SeD mice. Squares represent males and circles represent females within each treatment group. Data were analyzed by two-way ANOVA with Tukey’s post-hoc analysis. Significance is indicated within the figure: ** *p*< 0.01, **** *p* < 0.0001, n.s., non-significant. For western blots, *n* = 7; for activity measurements, *n* = 8.

Gpx2 and Gpx4 are highly expressed in lung tissues. Thus, protein levels and activities were measured in lung homogenates generated from SeS and SeD pups (Figure 3a–c). An independent effect of SeD on Gpx protein levels was observed. Notably, Gpx2 and Gpx4 proteins were barely detectable in SeD pup tissues compared to SeS pups. In SeS pups, hyperoxia exposure increased the levels of both Gpx2 and Gpx4 protein. In contrast, lung Gpx2 and Gpx4 levels were not different between RA and hyperoxia-exposed SeD pups (a and b). Enzymatic activity was impacted by both protein abundance as well as catalytic function. Thus, we measured the total Gpx activity in lung homogenates. Se deficiency independently impacted lung Gpx activity, which was greatly reduced compared to SeS pups. Our data revealed a modest effect of hyperoxia exposure on Gpx activity in both SeS and SeD groups (c).

### 3.4. Selenoprotein Expression

**Figure 4:**
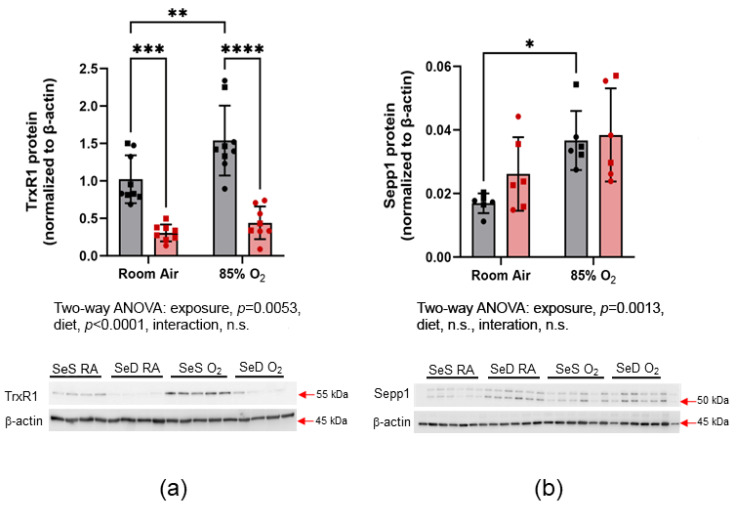
Selenoprotein expression. Txnrd1 (**a**) and Sepp1 (**b**) protein levels were measured by western blot analysis. Grey bars with black symbols represent SeS mice while red bars and red symbols represent SeD mice. Squares represent males and circles represent females within each treatment group. Data were analyzed by two-way ANOVA, with Tukey’s post-hoc analysis. Significance is indicated within the figure: * *p* < 0.05, ** *p*< 0.01, *** *p* < 0.0005, **** *p* < 0.0001, n.s., non-significant. For Txnrd1, *n* = 9 in the SeS group and *n* = 8 in the SeD groups. For Sepp1, *n* = 6 for all groups.

To further characterize the putative selenoprotein hierarchy, selenoprotein P (SelenoP) and thioredoxin reductase (Txnrd1) were measured by western blot analysis, to determine the effects of Se deficiency on non-Gpx, Se-dependent proteins (Figure 4a,b). Txnrd1 protein levels in SeS pups were greater following 85% O_2_ exposure than RA exposure (a). Txnrd1 protein levels were dramatically reduced in SeD pups and, again, no effect of hyperoxia was detected. SelenoP levels were greater in the SeS pups exposed to 85% O_2_ compared to room-air-exposed SeS pups. No differences were observed between SeD pups raised under RA or hyperoxia (b).

### 3.5. Nrf2 Activation

**Figure 5:**
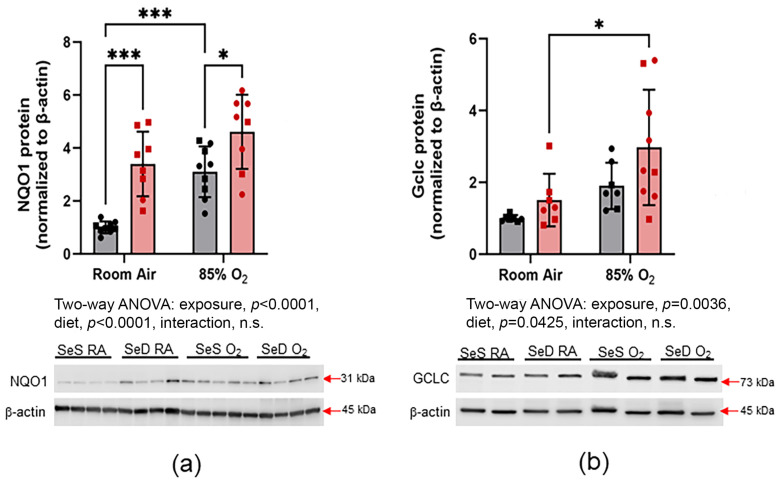
Nrf2 activation. NQO1 (**a**) and Gclc (**b**) protein levels were measured by western blot analysis, as indicated in Methods. Grey bars with black symbols represent SeS mice while red bars and red symbols represent SeD mice. Squares represent males and circles represent females within each treatment group. Data were analyzed by two-way ANOVA with Tukey’s post-hoc analysis. Significance is indicated within the figure: * *p* < 0.05, *** *p* < 0.0005, n.s., non-significant. For NQO1, *n* = 9 in the SeS group and *n* = 8 in the SeD group. For Gclc, *n* = 7 in the SeS group and for SeD/RA, *n* = 9 in the SeD/O_2_ group.

Nrf2 target gene expression has been shown to increase in settings of hyperoxia exposure and Se deficiency (Figure 5). NQO1 transcription is directly regulated by Nrf2, and NQO1 protein levels are often used as a surrogate for Nrf2 activation. In these studies, SeD pups had a greater basal protein expression of NQO1 than SeS pups. Exposure to 85% O_2_ increased NQO1 expression in SeS pups. Notably, hyperoxia did not increase NQO1 expression in SeD pups (a). Glutamate-cysteine ligase catalytic subunit (Gclc) is also transcriptionally regulated by Nrf2 and is an essential component of de novo GSH synthesis. In SeS pups, no differences were observed in Gclc protein levels between RA and 85% O_2_-exposed pups. In contrast, hyperoxia exposure increased Gclc expression in lung tissues from SeD pups (b).

### 3.6. Lung RNA-Seq Analyses

**Figure 6:**
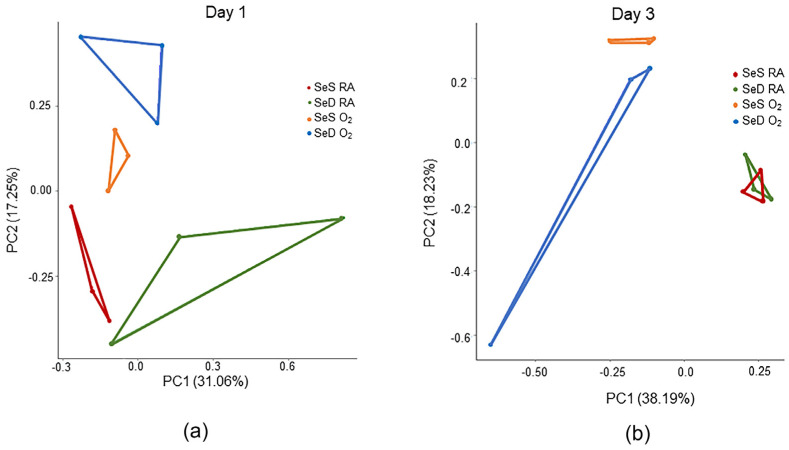
Principal component analysis (PCA) plots. (**a**) PCA plot from RNA-seq analysis of PN1 lung tissues from SeS or SeD pups exposed to RA or hyperoxia. (**b**) PCA plot from RNA-seq analysis of PN3 lung tissues from SeS or SeD pups exposed to RA or hyperoxia. Complete separation among the four groups is observed at PN1, and the O_2_ groups were separated from each other and the RA groups at PN3.

To understand early changes elicited by SeD and/or hyperoxia exposure that may contribute to observed morphologic findings in our study, RNA was isolated from the lung tissues obtained from SeS and SeD pups exposed to RA and O_2_ for 24 h (PN1) or 72 h (PN3). This early time point was chosen to identify the instigating events that would lead to the phenotype observed at PN14. Isolated RNA was analyzed by bulk RNA sequencing. Results revealed 2816 differentially expressed genes (DEGs) between SeS and SeD in RA, 782 DEGs between SeS and SeD in O_2_, and 381 DEGs at the intersection between Se status and O_2_ exposure (Table 1). Principle component analyses revealed four separated groups at PN1, and the O_2_ groups were separated from each other and the RA groups at PN3 (Figure 6).

### 3.7. GSEA Hallmark Analyses Revealed Differences within the Context of Se Deficiency and Hyperoxia Exposure

**Figure 7:**
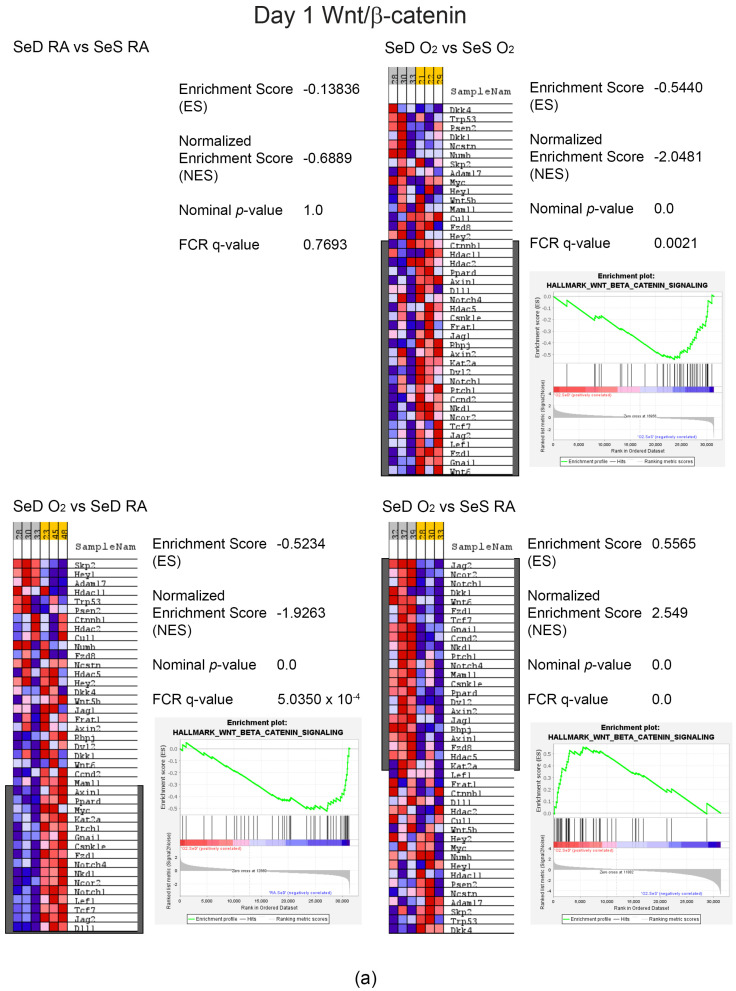

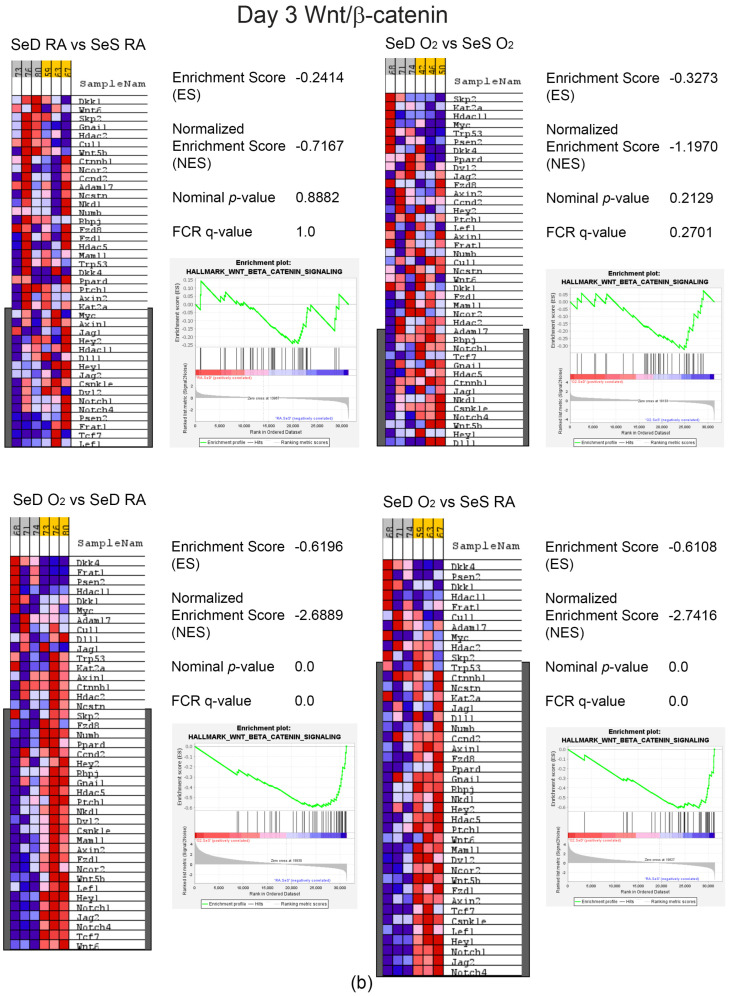
GSEA core enrichment analyses of Wnt/β-catenin pathway. Heat maps and enrichment plots were produced on the Wnt/b-catenin pathway for the following group comparisons: SeD RA vs. SeS RA, SeD O_2_ vs. SeS O_2_, SeD O_2_ vs. SeD RA and SeD O_2_ vs. SeS RA on Day 1 (**a**) or Day 3 (**b**). Grey boxes indicate “yes” in core enrichment. Statistics are indicated in the figures.

**Figure 8:**
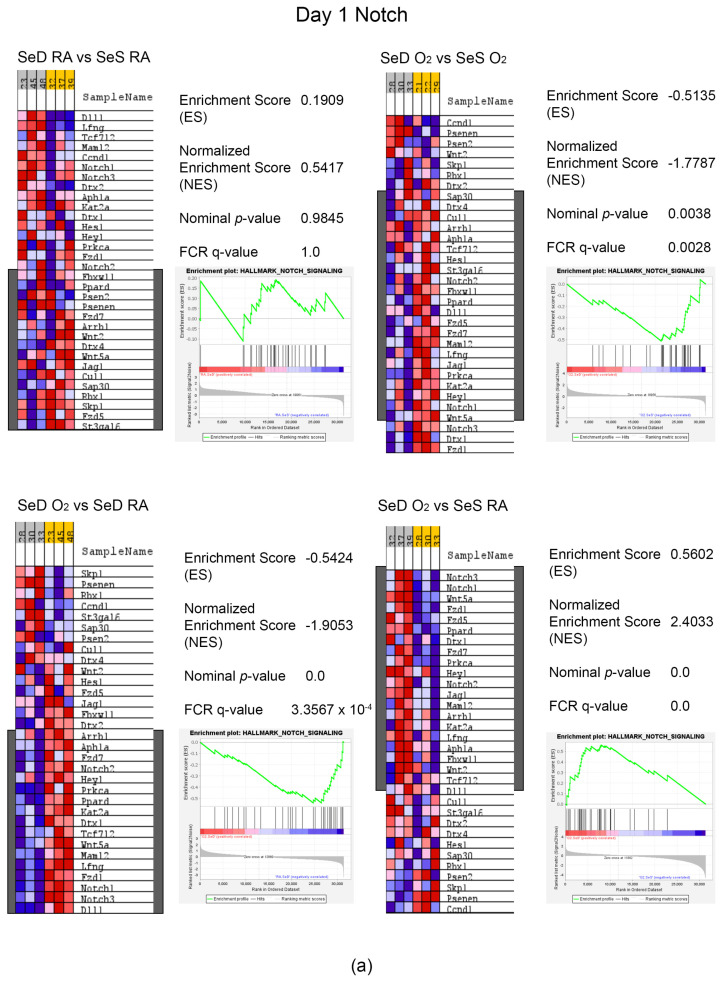

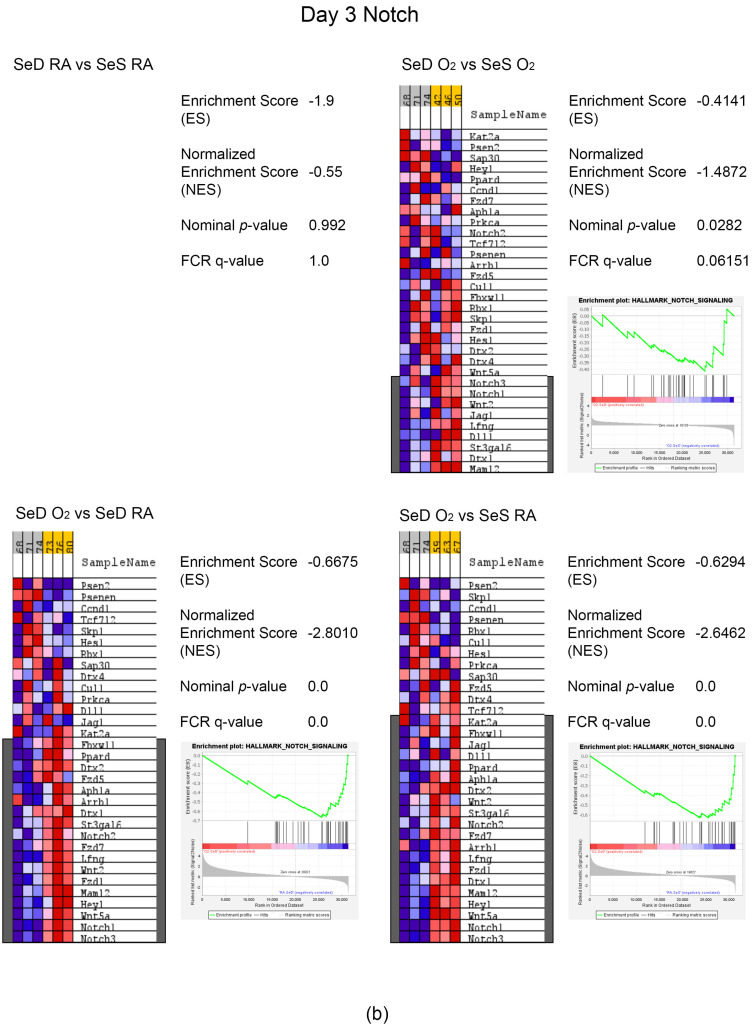
GSEA core enrichment analyses of Notch pathway. Heat maps and enrichment plots were produced on the Notch pathway for the following group comparisons: SeD RA vs. SeS RA, SeD O_2_ vs. SeS O_2_, SeD O_2_ vs. SeD RA and SeD O_2_ vs. SeS RA on Day 1 (**a**) or Day 3 (**b**). Grey boxes indicate “yes” in core enrichment. Statistics are indicated within the figures.

As expected, differences were seen in the oxidative stress and redox pathway, glutathione metabolism, and selenium metabolism (Supplemental Figure S1). Intriguingly, pathways directly associated with lung development were differentially expressed and included Notch and WNT/b-catenin signaling. Enrichment analysis of the Wnt/β-catenin pathway resulted in core enrichment genes including Wnt5b, Wnt6, Axin2 and Frizzled1 and 8 (Figure 7a,b). Notch core enrichment genes included Notch 1, 2 and 3, Jagged 1 (Jag1), and Delta 1 (Dl1) (Figure 8a,b). Overall, core enrichment genes in both pathways were suppressed in SeD pups exposed to hyperoxia compared to SeD in RA or SeS in either exposure group (Figure 7 and Figure 8).

## 4. Discussion

Oxygen toxicity and antioxidant deficiencies contribute to the development of BPD. Se deficiency is common in premature infants, exacerbates antioxidant deficiencies and is correlated with BPD development [4,28]. ELBW infants are at even greater risk of Se deficiency and the development of BPD because of their early birth. The mechanisms by which Se status influences neonatal lung development are unknown. Earlier animal studies identified effects of Se deficiency in lung growth and development in calves [29] and rats [30]. Hawker et al. [31] characterized the effects of hyperoxia exposure in the same rat model and Kim et al. demonstrated the beneficial effects of Se repletion on lung development in hyperoxia-exposed newborn rats [32]. Building upon our previous study in Se-deficient newborn C57Bl/6J mice, which demonstrated impaired lung development and alveolarization [33], the present investigation indicates that the effects of Se status are strain-independent. Our data further supports the finding that Se status independently modifies perinatal lung development and establishes an exaggerated phenotype under hyperoxia in C3H/HeN mice.

In the present study, litter sizes and weights were not different at birth through to PN14 between C3H/HeN SeD and SeS pups. These data differ from our previously reported findings in the C57Bl/6J strain, in which we observed modestly lower weights in SeD pups, beginning at PN7 and persisting through adulthood [33]. Se-dependent deficits in lung development were similar in both strains of mice. Our interpretation of these data is that the C57Bl/6 was more sensitive to Se deficiency in overall growth, for reasons which are not readily apparent.

To test the hypothesis that Se deficiency would exacerbate hyperoxia-induced lung injury, we incorporated our murine model of BPD, with pups exposed to 85% O_2_ for 14 d. More than 40% of the SeD pups exposed to hyperoxia died during this time (Figure 1), demonstrating that perinatal Se deficiency has a substantial effect on the viability of the pups, likely due to overwhelming oxidative stress as reflected in the early alterations in redox and oxidative stress pathways revealed in our RNA-seq analyses. Lung growth and structure were altered by Se deficiency alone. As noted previously in C57Bl6/J mice, alveolar size was larger and alveolar numbers lower in SeD pups exposed to RA compared to SeS pups exposed to RA. Hyperoxia exposure exacerbated SeD-associated deficits, creating a more underdeveloped lung with interrupted alveolarization. We interpret these findings to suggest that Se, either directly or indirectly through selenoproteins, modulates pathways that govern lung development (Figure 2).

Present in millimolar levels, the GSH system is the primary antioxidant system in mammalian cells and includes isoforms of Gpx and the GSH-synthesizing enzyme, GCLC. Gpx 2 and 4 isoforms are highly expressed in lung epithelia and play a significant role in detoxifying oxidant stress due to inhalation exposures [34,35]. In settings of oxidative stress, Nrf2 is activated, facilitates the upregulation of de novo GSH synthesis through *Gclc* induction, and enhances GSH system function through increased *Gpx* activity. Consistent with established antioxidant responses, both Gpx2 and Gpx4 levels were increased in lungs from SeS pups exposed to hyperoxia. As would be expected in settings of Se deficiency, both Gpx 2 and Gpx4 levels were profoundly lower in SeD pups. Concomitant decreases in pulmonary Gpx activity in SeD pups suggest the presence of compromised GSH-dependent antioxidant capacity in SeD pups (Figure 3), likely driven by both decreased expression and compromised catalytic activity due to the absence of active site Sec. Gclc protein levels were increased in SeD pups exposed to 85% O_2_; however, no increase was observed in lungs from hyperoxia-exposed SeS pups (Figure 5). This finding could suggest an attempt to upregulate de novo GSH synthesis to protect the lung from oxidant stress caused by hyperoxia exposure. The lack of enhanced Gclc expression in SeS pups exposed to hyperoxia at 14 d does not preclude an induction of GSH-dependent responses at earlier time points, as observed in our previous studies [22].

A weakness of our study is that our results could also be influenced by the unusually high mortality rates observed in the hyperoxia-exposed SeD pups: our measurements were made on pups able to overcome the significant oxidant stress of diet and exposure. The high mortality observed in hypoxia-exposed SeD pups may represent a subset of pups unable to mount a sufficient antioxidant response to overcome the oxidant stress imposed. However, we cannot rule out altered cardiorespiratory responses or severe neurological dysfunction, as previously observed as the cause of death in our pups [16].

The Trx antioxidant system is present in micromolar amounts, and its function is largely dependent on the enzymatic activity of the selenoprotein Txnrd1. The impact of the Trx system disruption is less likely to impact antioxidant responses and more likely to alter signaling pathways associated with lung morphogenesis [36]. As with Gpx, Txnrd1 protein expression was increased in lungs from hyperoxia-exposed SeS pups. In contrast, SeD pups demonstrated substantially lower pulmonary Txnrd1 protein levels, and hyperoxia exposure had no impact on expression (Figure 4). SelenoP is an essential component of Se-dependent processes as the primary means for Se transport throughout the body. As might be expected in a setting of oxidant stress, the observed increase in SelenoP protein levels in lungs from hyperoxia-exposed SeS pups likely occurs to enhance systemic Se transport and delivery to support increases in Se-dependent antioxidant responses. In contrast, SelenoP levels were not enhanced by hyperoxia exposure in SeD pups. This is suggestive of impairments in antioxidant responses under Se deficiency. In this context, enhanced mortality and hyperoxia lung injury in SeD pups would be expected. The overall trends on Gpx2, Gpx4, Txnrd1 and SelenoP expression were greater under SeD conditions in RA-exposed mice, suggesting the presence of a lung selenoprotein hierarchy of SelenoP > Txnrd1 > Gpx2/Gpx4.

Previous studies from our group have established a relationship between SeD and attenuated Nrf2-dependent responses [11]. Further, the genetic enhancement of basal Nrf2-mediated antioxidant responses decreases the effects of O_2_ toxicity on perinatal lung development in a murine model of BPD [37]. NQO1 protein levels were measured as a surrogate of Nrf2 activation. NQO1 protein expression was elevated in SeD pup lung tissues. We interpret this finding to indicate basal upregulation of Nrf2-dependent antioxidant responses in the context of Se deficiency, which is consistent with previous findings (Figure 5) [11]. NQO1 protein was greater in hyperoxia-exposed pups than in RA-exposed pups, supporting a further induction of Nrf2-responsvie genes in the oxidant environment of hyperoxia. In contrast, under SeD conditions, additional Nrf2 induction was not apparent and likely contributed to the morality and altered lung development observed in the present studies.

Beyond proteins involved in antioxidant responses, Nrf2 is known to regulate many processes responsible for normal lung development [38]. Thus, we further speculate that Se is a clinically relevant modulator of similar pathways involved in lung development [38]. Consequently, we performed RNA sequencing analyses on lung tissues from all groups, to identify pathways that might intersect with Se deficiency and hyperoxia exposure. We performed these analyses at early time points in lung development and hyperoxia exposure (PN1 and PN3), to elucidate early effects of perinatal SeD and hyperoxia that may be responsible for our observed findings at 14 d. The GSEA Hallmark database was interrogated for changes in pathways due to Se deficiency and hyperoxia exposure (Supplemental Figure S1). As would be expected, differential expression was observed in the “Reactive Oxygen Species Pathway” under increases in the oxygen-exposed pups. More interesting and relative to lung development are the differential expression profiles in Wnt/β-catenin [39] and Notch pathways [40,41,42]. Enrichment analyses were further performed on these specific pathways (Figure 7 and Figure 8).

Enrichment analyses of the Wnt/b-catenin pathway identified several Wnt signaling molecules, including Axin1 and several Fzd isoforms at PN1. PN3 revealed suppression of Wnt5b and Wnt6. Wnt5b has been identified as a negative regulator of alveolar epithelial progenitor cells, thus restricting growth and differentiation in the alveolar compartment [43]. Wnt6 is influential earlier in development, playing a role in embryonic morphogenesis and later in postnatal homeostasis (reviewed by Wei) [44]. Lower expression of these Wnt/b-catenin pathway genes could significantly influence lung development and are evident in the context of Se deficiency and hyperoxia exposure.

Enrichment analyses of the Notch pathway identified several canonical genes. Notch 1, 2 and 3 exhibit lower expression in hyperoxia and under Se deficiency. Notch 2 has been identified as crucial to alveolar epithelial differentiation and the subsequent integrity of the epithelial and smooth muscle layers of the distal conducting airways. Notch 1 works in conjunction with Notch 2 in epithelial maintenance but is not essential for lung development [45]. Notch 3 regulates smooth muscle cell differentiation and distribution as well as club cell and basal cell differentiation into epithelial cells in the lung. Importantly, Notch ligands are also differentially expressed in our models and include Jag1 and Dll1. Both are important ligands for Notch 2 and 3 and are essential for developmental signaling in the lung, though the cell types expressing the ligand are not well defined.

Interestingly, both Wnt/β-catenin and Notch signaling are associated with Nrf2. Wnt3a has been shown to stabilize Nrf2 in fibrotic lung diseases and alter antioxidant metabolism in hepatocytes [46,47]. The interactions between Notch signaling and Nrf2 are reasonably well-defined and include reciprocal regulation. Notch has an antioxidant binding element (ARE, the Nrf2 binding site) in the promoter region and Nrf2 has a Rbpjk site (the Notch binding site) in its promoter region. This reciprocal regulation was defined by Wakabayashi et al. in the context of hepatic oxidant stress [42] for Notch1. Other studies have identified a ROS-Nrf2-Notch cellular homeostasis that prevents airway diseases associated with oxidant stress [40,41].

## 5. Conclusions

Our data suggest that Se is a biologically relevant modulator of perinatal lung development and antioxidant responses, especially in the context of hyperoxia exposure. Further, our data highlight that beyond antioxidant effects, relevant pulmonary genes and pathways implicated in the development of BPD are differentially modulated by the intersection of perinatal Se status, Nrf2 signaling and hyperoxia in the neonatal lung. Given the enhanced risk of Se deficiency in ELBW infants, the current data represent a viable model to identify the mechanistic role of Se in perinatal lung development and hyperoxia responses. Additional information provided by RNA sequencing data will open new avenues to identify lung developmental pathways dysregulated by Se deficiency, as well as the interaction between Se deficiency and O_2_ toxicity.

## Figures and Tables

**Table 1:** Differentially expressed genes from RNA seq.

Significant Genes in Each Comparison			
O_2_ vs. RA in SeS	O_2_ vs. RA in SeD	SeS vs. SeD in RA	SeS vs. SeD in O_2_
6813	4239	2816	782

Significant genes were selected from filtered normalized 31420 genes by using the Wald test with Deseq2 in R. Threshold setting: false discovery rate (FDR adj. *p*) < 0.05; log fold change > |0.25|.

## Data Availability

The transcriptomic data presented in this study are available in the GEO database (accession number GSE255744). Public accessibility information: Transcriptomic data will be accessible on 13 February 2025.

## Supplementary Materials

The following supporting information can be downloaded at https://www.mdpi.com/article/10.3390/antiox13040391/s1: Figure S1: GSEA Hallmark Gene Sets.
