# Supplementary figures and images for: Selenium Deficiency Exacerbates Hyperoxia-Induced Lung Injury in Newborn C3H/HeN Mice
Source: Antioxidants (Basel). 2024 Mar 25;13(4):391. doi: 10.3390/antiox13040391 (PMC11047402; doi:10.3390/antiox13040391)

# GSEA Hallmark Gense sets (All comparison)

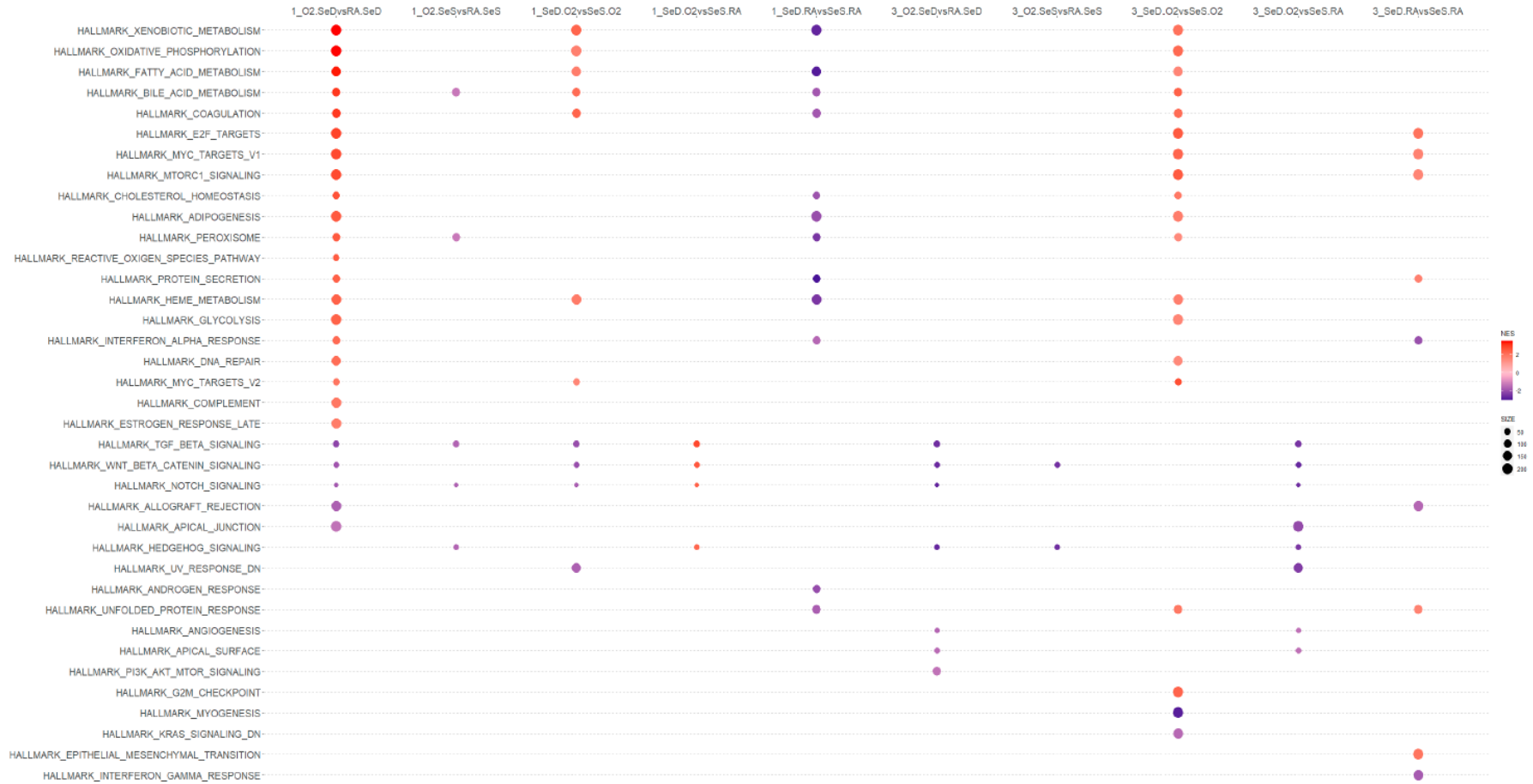

Figure S1. GSEA Hallmark Gense Sets.

Supplement: Supplementary file 1 [file antioxidants-13-00391-s001.zip › antioxidants-2893270-supplementary.pdf]
